# Supplementary material for: Validation and Adaptation of the Multidimensional Prognostic Index in an Older Australian Cohort
Source: J Clin Med. 2019 Nov 1;8(11):1820. doi: 10.3390/jcm8111820 (PMC6912422; doi:10.3390/jcm8111820)
Supplement: Supplementary file 1 [file jcm-08-01820-s001.pdf]

supplementary RESULTS

Table 1. Baseline characteristics according to follow-up status.

	Completed follow-up	Lost to follow-up	*p* value
	n = 697	*n* = 40	
Age, years (IQR)	80 (72–86)	79 (72-84)	0.5294
Age range	65–102	65 – 96	
Female, n (%)	349 (50.07)	21 (52.50)	
AMT, score (IQR)	9 (8–10)	9 (8–10)	0.6708
ARS, score (IQR)	0 (0–2)	0 (0–1)	0.0746
RUDAS, score (IQR)	26 (24–28)	27 (23.5–28.5)	0.3796
MPI, score (IQR)			
ADL	6 (5–6)	6 (5–6)	0.1612
IADL	6 (3–7)	7 (5–8)	0.0069
SPMSQ	1 (0–1)	1 (0.5–2)	0.6577
ESS	18 (16–19)	18 (17–19)	0.6452
CIRS-CI	7 (5–8)	6 (4–7)	0.0039
CIRS-SI	2.54 (2.23–2.77)	2.27 (2.08–2.58)	0.0162
MNA	20.5 (17.5–23.0)	22.5 (20–24.5)	0.0097
Number of drugs	9 (6–12)	7 (5–10)	0.0068
Cohabitation status	2 (1–2)	2 (1–3)	0.9722
Total MPI, score	0.436 (0.313–0.500)	0.313 (0.250–0.438)	0.0012
Fall, n (%)	20 (2.87)	1 (2.50)	0.8914
Delirium, n (%)	67 (9.61)	4 (10.00)	0.9357
LOS, in days (IQR)	6 (3–11)	5.5 (4–11)	0.9487
Mortality, n (%)			
In-hospital	25 (3.59)	0 (0.00)	–
1-month	35 (5.02)	–	–
3-month	65 (9.33)	–	–
6-month	137 (19.66)	–	–
12-month	201 (28.84)	–	–
Re-admission rate, %			
1-month	13.63	–	–
3-month	29.56	–	–
6-month	41.75	–	–

Note: results are median with interquartile range (IQR), unless otherwise stated. Abbreviations: AMT: Abbreviated Mental Test; ADL: Activity of daily living; ARS: Anticholinergic risk scale; CIRS-CI: Cumulative illness rating scale-illness severity score; CIRS-CI: Cumulative illness rating scale-comorbidity index; ESS: Exton-Smith scale; IADL: Instrumental activities of daily living; LOS: length of stay; MNA: Mini nutritional assessment; MPI: Multidimensional prognostic index; n: number; RUDAS: Rowland University dementia assessment score; SPMSQ: Short portable mental status questionnaire.

Table 2. Unadjusted and Adjusted logistic regression for original MPI, adapted MPI, and 12-month all-cause mortality.

	Unadjusted OR			Adjusted OR*		
**Variable**	OR	95% CI	*p*-value	OR	95% CI	*p*-value
**Original MPI**						
MPI Mild	reference group			reference group		
MPI Moderate	2.50	1.67–3.75	<0.0001	2.48	1.64 – 3.77	<0.0001

MPI Severe	4.24	2.28–7.88	<0.0001	4.07	2.12–7.82	<0.0001
**Subgroups**						
Original MPI Continuous	2.17	1.62–2.90	<0.0001	2.13	1.57–2.90	<0.0001
**Adapted MPI (ARS)**						
MPI Moderate	2.46	1.74–3.49	<0.0001	2.58	1.80–3.70	<0.0001
MPI Severe	3.84	1.85–7.98	<0.0001	3.49	1.65–7.41	0.001
**Adapted MPI (RUDAS)**						
MPI Moderate	2.45	1.60–3.77	<0.0001	2.45	1.57–3.81	<0.0001
MPI Severe	4.68	2.56–8.55	<0.0001	4.40	2.33–8.32	<0.0001
**Adapted MPI (ARS & RUDAS)**						
MPI Moderate	2.09	1.47–2.98	<0.0001	2.13	1.48–3.06	<0.0001
MPI Severe	3.80	1.97–7.35	<0.0001	3.47	1.77–6.82	<0.0001

Abbreviations: ARS: Anticholinergic risk scale; CI: confidence interval; MPI: Multidimensional Prognostic Index; OR: Odds ratio; RUDAS: Rowland University dementia assessment score. Note: * adjusted for age and gender.

Table 3. FMC cohort hazard ratios for Original MPI risk categories.

	**Unadjusted HR**			**Adjusted HR***		
Variable	HR	95% CI	*p*-value	HR	95% CI	*p*-value
MPI Mild	reference group			reference group		
MPI Moderate	2.26	1.58 - 3.24	<0.0001	2.26	1.56 - 3.26	<0.0001
MPI Severe	3.65	2.23 - 5.98	<0.0001	3.72	2.22 - 6.24	<0.0001

Abbreviations: CI: confidence interval; HR: Hazard ratio; MPI: Multidimensional Prognostic Index. Note: * adjusted for age and gender.

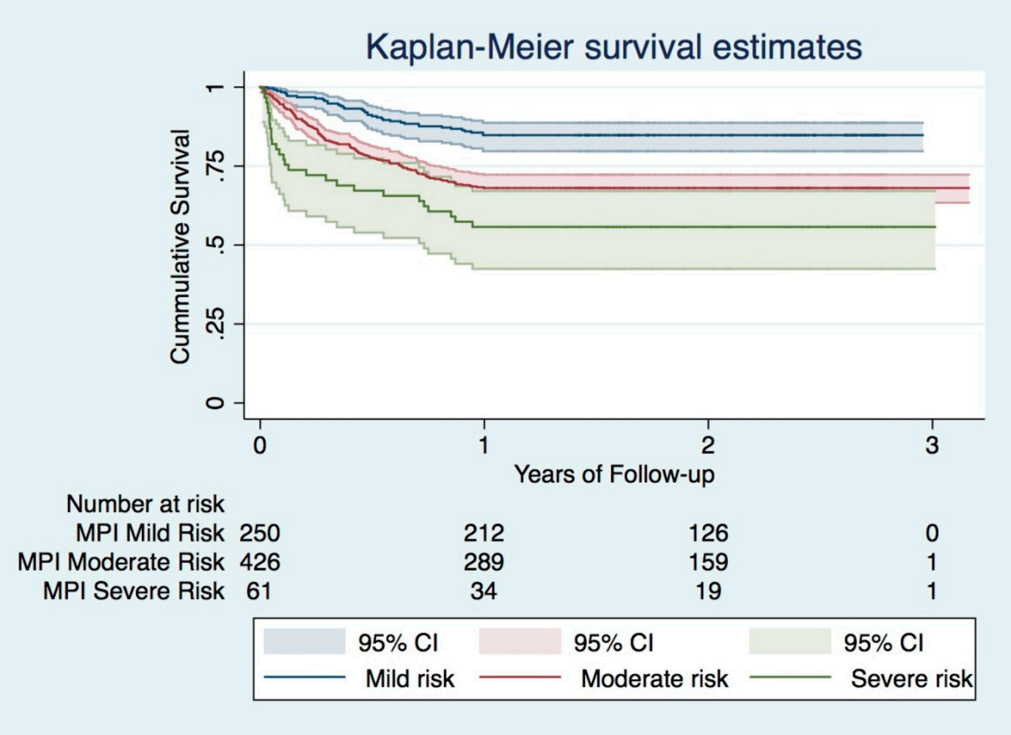

Figure 1. Survival curve for MPI risk categories.

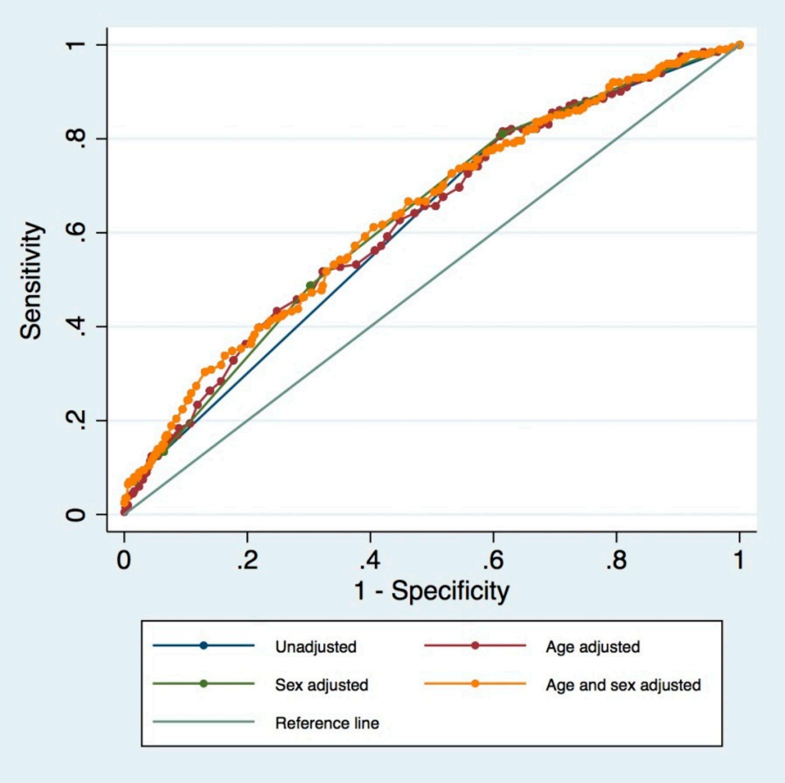

Figure 2. Unadjusted and adjusted ROC curves for original MPI and 12-month all-cause mortality.

Table 4. Area under the ROC curves for original and Adapted MPI's.

12-month All-Cause Mortality	Adjusted	AUC	95% CI
Original MPI		0.613	0.575–0.652
	Age	0.629	0.584–0.674

	Sex	0.633	0.589–0.676
	Age & Sex	0.639	0.594–0.684
MPI with substituted ARS		0.621	0.580–0.662
	Age	0.639	0.594–0.683
	Sex	0.645	0.600–0.690
	Age & Sex	0.647	0.602–0.693
MPI with substituted RUDAS		0.613	0.575–0.651
	Age	0.627	0.582–0.673
	Sex	0.629	0.586–0.672
	Age & Sex	0.636	0.591–0.681
MPI with substituted ARS and RUDAS		0.607	0.566–0.648
	Age	0.629	0.583–0.674
	Sex	0.625	0.580–0.670
	Age & Sex	0.633	0.587–0.679

Abbreviations: ARS: Anticholinergic risk scale; AUC: Area under ROC curve; CI: confidence interval; MPI: Multidimensional Prognostic Index; RUDAS: Rowland University dementia assessment score.
